# Special Issue “Human Picornaviruses”

**DOI:** 10.3390/v12010093

**Published:** 2020-01-13

**Authors:** Petri Susi

**Affiliations:** Institute of Biomedicine, University of Turku, 20520 Turku, Finland; pesusi@utu.fi

The Special Issue “Human Picornaviruses” in “*Viruses*” (Submission Deadline 30 September 2019, https://www.mdpi.com/journal/viruses/special_issues/Picornaviruses) includes twelve original articles and four reviews with a wide range of topics. Papers about virus recombination and recombinants identified by means of next-generation sequencing and bioinformatics, viral cell surface receptors, virus replication, interaction with host cells, and immune responses are included. While several papers (Osundare et al. [1], Chamings et al. [2], Vakulenko et al. [3] and Hietanen & Susi [4]) deal with generic amplification, sequencing, identification and/or typing of viral sequences and viruses, the second paper by Vakulenko et al. [5] is of major importance, as it deals with the effects of sample bias and experimental artefacts on the outcome of phylogenetic analyses. The results suggest that even a single erroneous sequence may profoundly destabilize the whole analysis by increasing the variance of the inferred evolutionary parameters. The paper gives suggestions on how to resolve the problems with less-than-optimal sequences. These data call for general guidelines for picornaviral sequence data analysis for optimal and correct data interpretation by means of phylogenetics. The paper by Li et al. [6] about picornaviral 2B protein summarizes the current knowledge and biological functions of the protein, emphasizing its potential new role as an antiviral target. While much of the antiviral research with human picornaviruses has focused on structural proteins and virus structure, non-structural replication proteins have obtained less attention. Considering the unique nature of many viral proteins, they are likely to be optimal targets for future antiviral development. Finally, several papers address human parechoviruses [1,2,7,8], which are common viruses and often detected in epidemiological surveys. Occasionally, these viruses are linked to serious disease. While being common viruses, the molecular research around these viruses is still in infancy and deserves more attention. Altogether, the papers in this special issue exemplify the diversity and importance of human picornaviruses as research study subjects. I wish to express my sincere thanks to all authors who contributed to the Special Issue “Human Picornaviruses”.
